# Supplementary material for: Warped Bayesian Linear Regression for Normative Modelling of Big Data
Source: Neuroimage. Author manuscript; Available in PMC 2022 Oct 6. (PMC7613680; doi:10.1101/2021.04.05.438429)
Supplement: Appendix [file EMS154600-supplement-Appendix.pdf]

bioRxiv preprint doi: https://doi.org/10.1101/2021.04.05.438429; this version posted April 6, 2021. The copyright holder for this preprint (which was not certified by peer review) is the author/funder, who has granted bioRxiv a license to display the preprint in perpetuity. It is made available under aCC-BY 4.0 International license.

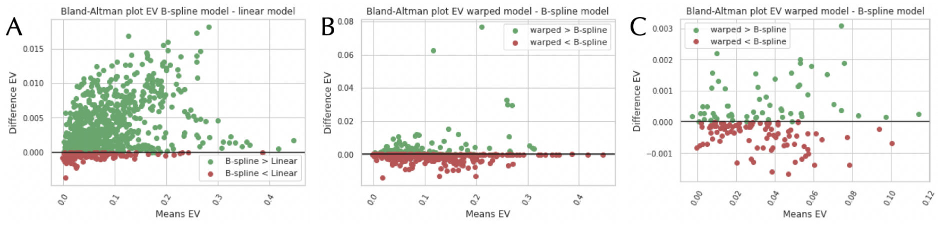

Figure A.8: Bland-Altman plots of the Explained Variance (EV): Figure A shows the comparison of the linear and B-spline model, using the IDPs. Figure B shows the comparison of the warped and B-spline model, using the IDPs. Figure C shows the comparison of the warped and B-spline model, using the FreeSurfer measurements.

Appendix A.

Figure A.8 shows the Bland-Altman plots of the explained variance for the IDPs and FreeSurfer measurements comparing the different model settings.

Appendix B.

An example list of the IDPs, processed using FUNPACK (the FMRIB UKBiobank Normalisation, Parsing And Cleaning Kit), used in this study is given in B.1. The IDPs contained the following neuroimaging modalities [17]:

1. T1, from which the total brain volumes are calculated.

bioRxiv preprint doi: https://doi.org/10.1101/2021.04.05.438429; this version posted April 6, 2021. The copyright holder for this preprint (which was not certified by peer review) is the author/funder, who has granted bioRxiv a license to display the preprint in perpetuity. It is made available under aCC-BY 4.0 International license.

Table B.1: Example list of the IDP field names, processed using FUNPACK (the FMRIB UKBiobank Normalisation, Parsing And Cleaning Kit).

Volumetric scaling from T1 head image to standard space
Volume of white matter
Median T2star in thalamus (left)
Mean FA in middle cerebellar peduncle on FA skeleton
Mean MD in middle cerebellar peduncle on FA skeleton
Mean MO in fornix on FA skeleton
Mean L1 in body of corpus callosum on FA skeleton
Mean L2 in cerebral peduncle on FA skeleton (right)
Mean L2 in cerebral peduncle on FA skeleton (right)
Mean OD in posterior limb of internal capsule on FA skeleton (right)
Mean ISOVF in splenium of corpus callosum on FA skeleton
Weighted-mean FA in tract acoustic radiation (left)
Weighted-mean MD in tract corticospinal tract (right)
Weighted-mean MO in tract acoustic radiation (right)
Weighted-mean L1 in tract acoustic radiation (left)
Weighted-mean L2 in tract acoustic radiation (left)
Discrepancy between T2 FLAIR brain image and T1 brain image
Volume of grey matter in Frontal Pole (left)

2.

Appendix C.

We computed the differences between the BICs of a B-spline BLR and a warped BLR. Afterwards, we selected the top 30 IDPs where the B-spline model had the lowest BIC comparatively to the warped score or the other

bioRxiv preprint doi: https://doi.org/10.1101/2021.04.05.438429; this version posted April 6, 2021. The copyright holder for this preprint (which was not certified by peer review) is the author/funder, who has granted bioRxiv a license to display the preprint in perpetuity. It is made available under aCC-BY 4.0 International license.

way around. In table C.2 the model selection criteria of the top 30 best-fitted IDPs with the B-spline BLR compared to the warped BLR are shown. In table C.3 the model selection criteria of the top 30 best-fitted IDPs with the warped BLR compared to the B-spline BLR shown. These tables demonstrate that every neuroimaging modality has its optimal model settings and that one should carefully examine the model selection criteria and shape of the response distribution, before choosing a model.

Appendix D.

We used a paired-sample t-test, pairing the IDP results (EV, MSLL and BIC) of the different models to estimate the difference between performance measures of the warped and non-warped BLR. In table D.4 and D.5 the Cohen's d effect sizes and p-values are reported. The results show that there is a large difference between the standard BLR and the B-spline BLR, which confirms that one should take into account the non-linearity of the data. For the warped BLR and the B-spline BLR model, there is only a significant difference in the BIC score. We argue that this is because the model selection criteria are not necessarily sensitive to the deviations in the residuals from normality. Therefore, we also recommend to, alongside the model selection criteria, look at the skewness and kurtosis values together with the QQ-plot to choose the optimal model settings for each modality.

Appendix E.

In table E.6 we listed the cognitive variables from the UK Biobank that were used in this study with their IDs.

bioRxiv preprint doi: https://doi.org/10.1101/2021.04.05.438429; this version posted April 6, 2021. The copyright holder for this preprint (which was not certified by peer review) is the author/funder, who has granted bioRxiv a license to display the preprint in perpetuity. It is made available under aCC-BY 4.0 International license.

EV	MSLL	BIC	Field
0.206	-0.115	-166562.002	Mean MD in superior fronto-occipital fasciculus on FA skeleton (right)
0.134	-0.072	-46220.575	Mean ISOVF in genu of corpus callosum on FA skeleton
0.025	-0.013	-12455.567	Mean MO in superior fronto-occipital fasciculus on FA skeleton (left)
0.159	-0.087	-163761.463	Mean L2 in superior fronto-occipital fasciculus on FA skeleton (right)
0.148	-0.08	-176269.475	Mean MD in external capsule on FA skeleton (right)
0.17	-0.093	-40955.602	Discrepancy between T1 brain image and standard-space brain template (linearly-aligned)
0.074	-0.039	-52218.319	Mean ISOVF in anterior limb of internal capsule on FA skeleton (left)
0.066	-0.034	-50151.283	Mean ISOVF in anterior limb of internal capsule on FA skeleton (right)
0.135	-0.072	-175704.326	Mean L3 in external capsule on FA skeleton (right)
0.202	-0.113	-32491.645	Mean ICVF in superior fronto-occipital fasciculus on FA skeleton (right)
0.077	-0.04	-99708.396	Inverted temporal signal-to-noise ratio in pre-processed tfMRI
0.188	-0.104	-171678.769	Mean MD in anterior corona radiata on FA skeleton (left)
0.265	-0.154	-176057.846	Weighted-mean MD in tract anterior thalamic radiation (left)
0.078	-0.041	-44211.387	Mean ISOVF in superior fronto-occipital fasciculus on FA skeleton (left)
0.143	-0.077	-59646.162	Weighted-mean ISOVF in tract anterior thalamic radiation (right)
0.177	-0.098	-172620.769	Mean MD in anterior corona radiata on FA skeleton (right)
0.273	-0.16	-176331.153	Weighted-mean MD in tract anterior thalamic radiation (right)
0.174	-0.096	-170432.707	Mean L2 in anterior corona radiata on FA skeleton (right)
0.054	-0.028	101219.506	Volume of grey matter in Pallidum (right)
0.175	-0.096	-169471.163	Mean MD in genu of corpus callosum on FA skeleton
0.229	-0.13	-175866.701	Weighted-mean L2 in tract anterior thalamic radiation (right)
0.163	-0.089	-177074.476	Mean MD in anterior limb of internal capsule on FA skeleton (left)
0.079	-0.041	-53234.386	Mean ISOVF in posterior corona radiata on FA skeleton (left)
0.159	-0.087	-58912.836	Weighted-mean ISOVF in tract anterior thalamic radiation (left)
0.04	-0.02	-25966.018	Mean ICVF in fornix on FA skeleton
0.076	-0.04	-56374.466	Mean ISOVF in anterior corona radiata on FA skeleton (left)
0.14	-0.075	-55319.609	Weighted-mean OD in tract superior thalamic radiation (left)
0.076	-0.039	-57122.197	Weighted-mean ISOVF in tract superior longitudinal fasciculus (left)
0.039	-0.02	-57205.686	Mean ISOVF in anterior corona radiata on FA skeleton (right)
0.103	-0.054	-51036.79	Mean ISOVF in posterior corona radiata on FA skeleton (right)

Table C.2: Model selection criteria of the top 30 IDPs, ranked according to difference between the BIC of a B-spline BLR and a SinhArcsinh warped BLR, where the B-spline BLR had a lower BIC score.

bioRxiv preprint doi: https://doi.org/10.1101/2021.04.05.438429; this version posted April 6, 2021. The copyright holder for this preprint (which was not certified by peer review) is the author/funder, who has granted bioRxiv a license to display the preprint in perpetuity. It is made available under aCC-BY 4.0 International license.

EV	MSLL	BIC	Field
0.249	-0.143	184900.524	Total volume of white matter hyperintensities (from T1 and T2-FLAIR images)
0.147	-0.079	-29710.013	Mean OD in fornix on FA skeleton
0.285	-0.164	-137192.133	Mean MD in fornix on FA skeleton
0.276	-0.153	-136161.29	Mean L3 in fornix on FA skeleton
0.275	-0.151	-134595.545	Mean L2 in fornix on FA skeleton
0.153	-0.083	-87376.141	Inverted temporal signal-to-noise ratio in pre-processed rfMRI
0.27	-0.157	-24636.152	Mean FA in fornix on FA skeleton
0.171	-0.093	-32985.173	Mean MO in anterior limb of internal capsule on FA skeleton (right)
0.094	-0.049	-22330.216	Mean MO in tapetum on FA skeleton (left)
0.043	-0.022	-26681.768	Mean MO in tapetum on FA skeleton (right)
0.141	-0.076	-33305.028	Mean MO in anterior limb of internal capsule on FA skeleton (left)
0.054	-0.027	-42459.737	Weighted-mean ISOVF in tract parahippocampal part of cingulum (left)
0.117	-0.062	-71451.215	Mean OD in splenium of corpus callosum on FA skeleton
0.064	-0.033	-40476.534	Weighted-mean FA in tract parahippocampal part of cingulum (right)
0.307	-0.183	-15506.712	Mean ISOVF in fornix on FA skeleton
0.182	-0.1	-34039.973	Discrepancy between T2 FLAIR brain image and T1 brain image
0.047	-0.024	-41660.315	Weighted-mean FA in tract parahippocampal part of cingulum (left)
0.058	-0.03	-51125.932	Mean OD in tapetum on FA skeleton (left)
0.199	-0.111	-172072.977	Weighted-mean MD in tract posterior thalamic radiation (left)
0.311	-0.186	-26746.982	Discrepancy between tfMRI brain image and T1 brain image
0.131	-0.071	-169248.259	Mean MD in posterior thalamic radiation on FA skeleton (left)
0.089	-0.046	-181090.417	Mean MD in inferior cerebellar peduncle on FA skeleton (left)
0.07	-0.036	-41654.584	Weighted-mean ISOVF in tract parahippocampal part of cingulum (right)
0.028	-0.014	-35788.551	Mean MO in posterior limb of internal capsule on FA skeleton (right)
0.069	-0.036	-62423.772	Weighted-mean OD in tract forceps major
0.027	-0.014	-52538.461	Mean ISOVF in middle cerebellar peduncle on FA skeleton
0.314	-0.188	-27837.003	Discrepancy between rfMRI brain image and T1 brain image
0.085	-0.044	-170720.346	Weighted-mean MD in tract medial lemniscus (right)

Table C.3: Model selection criteria of the top 30 IDPs, ranked according to the difference between the BIC of a B-spline BLR and a SinhArcsinh warped BLR, where the SinArcsinh warped BLR had a lower BIC score.

bioRxiv preprint doi: https://doi.org/10.1101/2021.04.05.438429; this version posted April 6, 2021. The copyright holder for this preprint (which was not certified by peer review) is the author/funder, who has granted bioRxiv a license to display the preprint in perpetuity. It is made available under aCC-BY 4.0 International license.

Criteria	t	p	d
EV	27.511	$p < 0.001$	0.922
MSLL	-26.538	$p < 0.001$	-0.889
BIC	-15.95	$p < 0.001$	-0.534

Table D.4: Table presenting a paired-sample t-test between the B-spline and standard BLR models, using the IDP data, showing a significant difference between the model selection criteria of the B-spline BLR and the standard BLR, with a large effect size.

Criteria	t	p	d
EV	-0.897	0.37	-0.03
MSLL	0.026	0.979	0.001
BIC	9.279	$p < 0.001$	0.311

Table D.5: Table presenting a paired-sample t-test between the B-spline and warped BLR models, using the IDP data, showing only a significant difference between the model selection criteria of the B-spline BLR and the B-spline SinhArcsinh warped BLR using the BIC score, with a small effect size.

bioRxiv preprint doi: https://doi.org/10.1101/2021.04.05.438429; this version posted April 6, 2021. The copyright holder for this preprint (which was not certified by peer review) is the author/funder, who has granted bioRxiv a license to display the preprint in perpetuity. It is made available under aCC-BY 4.0 International license.

Table E.6: Cognitive variables of the UK Biobank that were used in this study.

Field	FieldID
Number of times snap-button pressed	403
Duration to first press of snap-button in each round	404
Mean time to correctly identify matches	20023
Time elapsed	4256
Digits entered correctly	4259
Number of rounds of numeric memory test performed	4283
Time to complete test	4285
Duration screen displayed	4290
Number of attempts	4291
Prospective memory result	20018
Fluid intelligence score	20016
Number of fluid intelligence questions attempted within time limit	20128
Duration to complete numeric path (trail 1)	6348
Total errors traversing numeric path (trail 1)	6349
Duration to complete alphanumeric path (trail 2)	6350
Total errors traversing alphanumeric path (trail 2)	6351
Errors before selecting correct item in numeric path (trail 1)	6770
Errors before selecting correct item in alphanumeric path (trail 2)	6771
Interval between previous point and current one in numeric path (trail 1)	6772
Interval between previous point and current one in alphanumeric path (trail 2)	6773
Number of puzzles correctly solved	6373
Number of puzzles viewed	6374
Number of puzzles correct	6382
Number of puzzles attempted	6383
Number of puzzles correct	21004
Number of symbol digit matches attempted	23323
Number of symbol digit matches made correctly	23324
